# Association between gut microbiota and diapause preparation in the cabbage beetle: a new perspective for studying insect diapause

**DOI:** 10.1038/srep38900

**Published:** 2016-12-09

**Authors:** Wen Liu, Yi Li, Shuang Guo, Han Yin, Chao-Liang Lei, Xiao-Ping Wang

**Affiliations:** 1 Hubei Insect Resources Utilization and Sustainable Pest Management Key Laboratory, College of Plant Science and Technology, Huazhong Agricultural University, Wuhan 430070, P R China

## Abstract

Gut microbiota mediate the nutritional metabolism and play important roles in human obesity. Diapausing insects accumulate large fat reserves and develop obese phenotypes in order to survive unfavorable conditions. However, the possibility of an association between gut microbiota and insect diapause has not been investigated. We used the Illumina MiSeq platform to compare gut bacterial community composition in nondiapause- (i.e. reproductive) and diapause-destined female cabbage beetles, *Colaphellus bowringi*, a serious pest of vegetables in Asia. Based on variation in the V3-V4 hypervariable region of 16S ribosomal RNA gene, we identified 99 operational taxonomic units and 17 core microbiota at the genus level. The relative abundance of the bacterial community differed between reproductive and diapause-destined female adults. Gut microbiota associated with human obesity, including *Bacteroidetes*, *Firmicutes*, and *Proteobacteria*, showed a good correlation with diapause. This association between gut microbiota and diapause in the cabbage beetle may open a new avenue for studying insect diapause, as well as developing a natural insect obesity model with which to explore the mechanisms responsible for human obesity.

Many animals, such as nematodes, crustaceans, fish and insects, have evolved diapause (a programmed arrest of development during specific life stages) to adapt to seasonal changes and survive unfavorable environmental conditions^1^,^2^. Diapause-destined individuals generally store large fat reserves for diapause maintenance and post-diapause development^1^,^3^,^4^. However, it remains unclear how lipid is accumulated during diapause preparation, an important stage before diapause itself^5^.

Reproductive diapause in insects is restricted to the adult stage and is characterized by arrested ovary development in females, increased stress tolerance and lipid accumulation during diapause preparation^6^,^7^,^8^. Conversely, non-diapausing, reproductive insects store more proteins and carbohydrates in the ovary during the pre-oviposition period^4^. Interestingly, fat storage in diapausing insects seems to coincide with obese phenotypes^9^ suggesting that studies of animal obesity may help understand diapause.

In recent years a growing body of evidence has demonstrated the close relationship between gut microbiota and obesity in mammals^10^. The human gut contains an immense number of commensal bacteria that collectively comprise a special metabolic ‘organ’ and plays an important role in regulating nutrition^11^,^12^. For example, by upregulating *de novo* fatty acid biosynthetic gene expression, and increasing lipoprotein lipase activity, the gut microbiota of mice can induce triglyceride storage in adipocytes leading to an obese phenotype^13^. In insects, a large number of bacteria also colonize the gut and modulate nutritional metabolism by providing digestive enzymes and vitamins that improve digestive efficiency^14^. For example, the gut microbiota of *Drosophila melanogaster* produce bioactive metabolites that regulate lipid absorption and storage^15^. Therefore, gut microbiota play a key role in the regulation of lipid storage in both mammals and insects. The obese phenotypes of diapausing insects raises the question of whether gut microbiota also play a role in lipid accumulation during diapause preparation.

To address this question, we investigated the association between gut microbiota and reproductive diapause preparation in the cabbage beetle, *Colaphellus bowringi*, a serious pest of vegetables in Asia^16^. The 4 day period post-eclosion (PE) is the pre-oviposition period of reproductive female *C. bowringi* and the diapause preparation period of diapause-destined females. Both types of female feed intensively during this period. Nutrients in reproductive females are mainly stored in the ovary in the form of proteins, while nutrients in diapause-destined females are primarily channeled to the fat body (the adipose and metabolic tissue of insects) in the form of triglycerides^4^. These two different patterns of nutrient distribution make an important contribution to subsequent reproduction or diapause^3^,^17^. The observation that both diapausing *C. bowringi* and obese humans accumulate lipids led us to suspect that gut microbiota, which have been found to mediate obesity in mammals^10^, may be involved in the regulation of nutritional metabolism in diapause-destined female adults. To address this question, we compared the community composition of the gut bacteria of nondiapause-destined (hereafter reproductive), and diapause-destined, female adults by sequencing the V3-V4 hypervariable region of the 16S ribosomal RNA gene. Our discovery of an association between gut microbiota and insect diapause preparation may open a new avenue for the study of insect diapause.

## Results and Discussion

### Richness and diversity of gut microbiota

**Figure 1:**
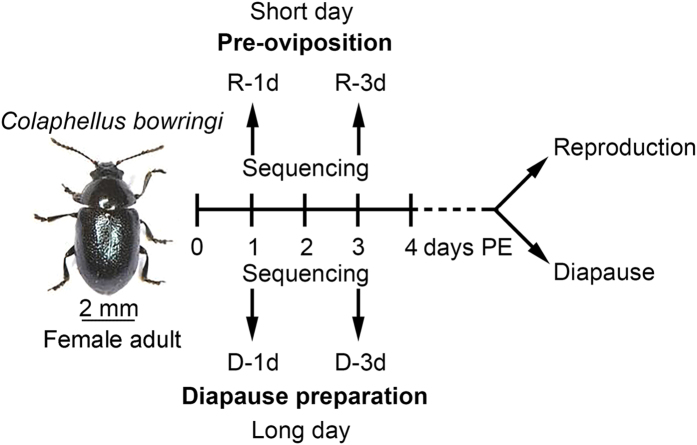
Schematic diagram of the experimental design used in this study. Reproductive females were obtained by raising larvae at 25 °C under a 12:12 h light:dark (LD) photoperiod and diapause-destined females by raising larvae at 25 °C under 16:8 LD photoperiod. Reproductive and diapause-destined female adults store energy obtained from plant foods in the ovary and the fat body, respectively, during the 4 day post-eclosion (PE) period. Gut microbiota were collected for sequencing from reproductive (R) and diapause-destined (D) females at 1 and 3 days PE. R-1d, reproductive adult at 1 day PE; R-3d, reproductive adult at 3 days PE; D-1d, diapause-destined adult at 1 day PE; D-3d, diapause-destined adult at 3 days PE.

**Figure 2:**
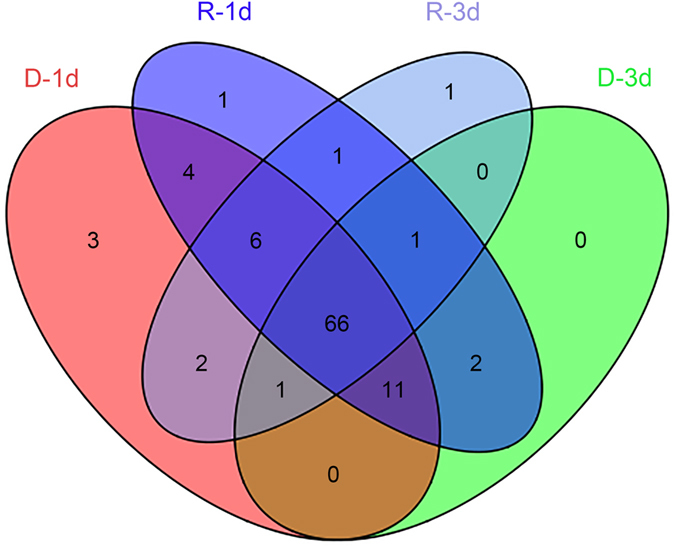
Venn diagram of OTU abundance in in reproductive (R) and diapause-destined (D) female cabbage beetles, *Colaphellus bowringi*, 1 and 3 days (d) post-eclosion.

To ask whether gut microbiota is associated with diapause, we compared the composition and diversity of the gut microbiota of reproductive and diapause-destined female *C. bowringi* at 1 and 3 days PE (Fig. 1). By sequencing the V3-V4 hypervariable region of the 16S ribosomal RNA gene and removing chimeric sequences and mismatches, we obtained 259,308 effective reads from four independent biological replicates of samples collected from reproductive (R-1d and R-3d) and diapause preparation (D-1d and D-3d) females (Fig. 1). Ninety-eight operational taxonomic units (OTUs) were identified (see Supplementary Table S1), of which 66 appeared in all samples (Fig. 2). After excluding the same taxonomic groups from these 66 OTUs, 17 core microbiota at the genus level were confirmed (Table 1). From the 98 OTUs, we identified 6 phyla, 11 classes, 21 orders, 35 families, 37 genera and 12 species. In detail, the 12 species were from the 10 identified genera, and the other 27 genera had not been annotated to species due to the very similar sequences of near-neighbor species. Nonetheless, these results indicate a high diversity of gut microbiota in both reproductive and diapause-destined female *C. bowringi*.

**Figure 3:**
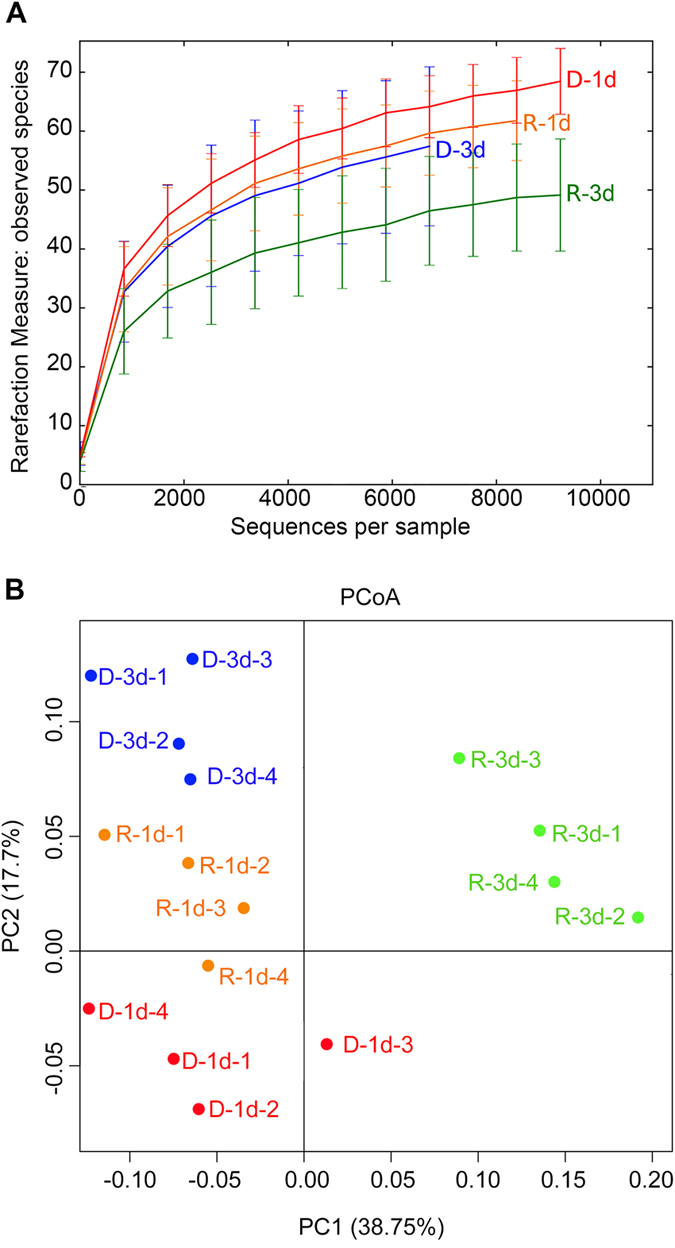
A rarefaction curve (**A**) and the Principal Coordinate Analysis (PCoA) analysis (**B**). The rarefaction curve and PCoA analysis, were generated from sequencing four independent biological replicates from each treatment with QIIME, and shows the rationality of sequencing data and the species abundance in each treatment. R-1d-1 represents the first independent biological replicate of R-1d, the others are deduced by analogy.

The number of microbiota species identified eventually plateaued, indicating that sufficient sequencing data had been obtained (Fig. 3A). After the normalization of data by referencing the minimum number of sequences (D-3d), the phase difference between D-1d and R-3d groups indicated an apparent difference (*P* = 0.032) in the diversity of gut microbiota between reproductive and diapause-destined females. In addition, the microbiota of both reproductive and diapause-destined females seem to have higher species diversity at 1d than at 3d PE although the differences were not significant (reproduction, *P* = 0.097; diapause, *P* = 0.362). Principal Coordinate Analysis (PCoA, weighted UniFrac) further indicated that the structure of gut microbiota in R-3d is different (ANOSIM *P* = 0.040) from D-1d (Fig. 3B). However, the structure of gut microbiota in reproductive (ANOSIM *P* = 0.090) or diapause (ANOSIM *P* = 0.100) individuals at 1d PE is similar to 3d PE. Hence, gut microbiota diversity and structure seems to be associated with reproductive status but not time PE. Therefore, it’s possible that the observed changes in the structure of the gut bacterial community of *C. bowringi* benefit either reproduction or diapause preparation.

### Taxonomy-based comparisons of gut microbiota

**Figure 4:**
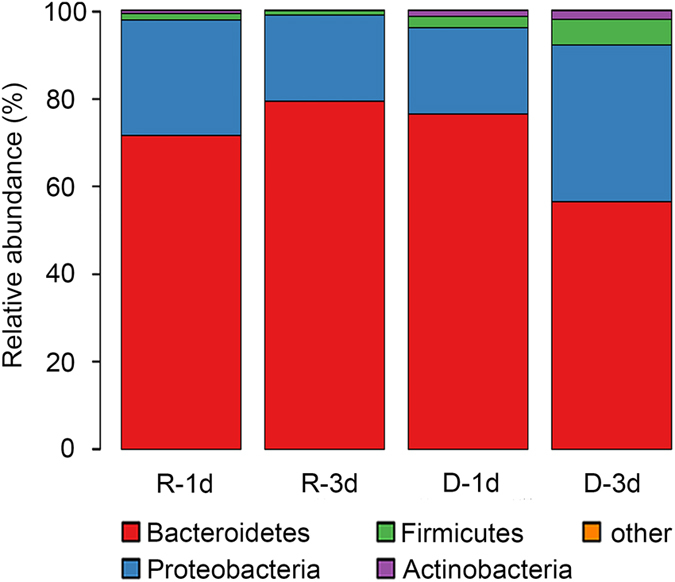
The relative abundance of gut microbiobial phyla in reproductive (R) and diapause-destined (D) female cabbage beetles, *Colaphellus bowringi*, 1 and 3 days (d) post-eclosion.

We analyzed the relative abundance of gut microbiota at different taxonomic levels in order to identify those that were reproductive and diapause-related. At the phylum level, *Bacteroidetes* and *Proteobacteria* were predominant (>98%), followed by the *Firmicutes* and *Actinobacteria* (Fig. 4). In the vertebrate gut, 80–90% of resident bacteria are *Bacteroidetes* and *Firmicutes*, followed by *Proteobacteria* and *Actinobacteria*^18^,^19^. This similar community composition indicates the conserved function of gut microbiota from invertebrates to vertebrates.

The abundance of *Bacteroidetes* was higher in the R-3d than in the D-3d group (*P* = 0.045), but the abundance of *Proteobacteria* (*P* = 0.049) and *Firmicutes* (*P* = 0.048) was lower in the R-3d than in the D-3d group (Fig. 4). There were no significant differences of the abundance of these bacteria between R-1d and D-1d groups (*P* > 0.05). These results suggest a positive correlation between the *Proteobacteria*, *Firmicutes* and diapause preparation, and a negative correlation between the *Bacteroidetes* and diapause preparation. Interestingly, a similar pattern has been reported in a study of mammalian obesity; compared to lean mice, obese mice underwent a 50% increase in the abundance of *Firmicutes* and a proportional reduction in that of *Bacteroidetes*^20^. Therefore, the accumulation of lipids in diapause-destined *C. bowringi*^4^ may be associated with an increase in human obesity-associated gut microbiota, such as *Firmicutes*. Some studies of rodents and humans have reached the opposite conclusion regarding the function of *Proteobacteria* in obesity^21^,^22^,^23^, but most available evidence suggests that *Proteobacteria* can regulate host obesity^24^. Therefore, we think it possible that the higher abundance of *Proteobacteria* in diapause-destined *C. bowringi* may be related to fat storage during diapause preparation^4^.

**Figure 5:**
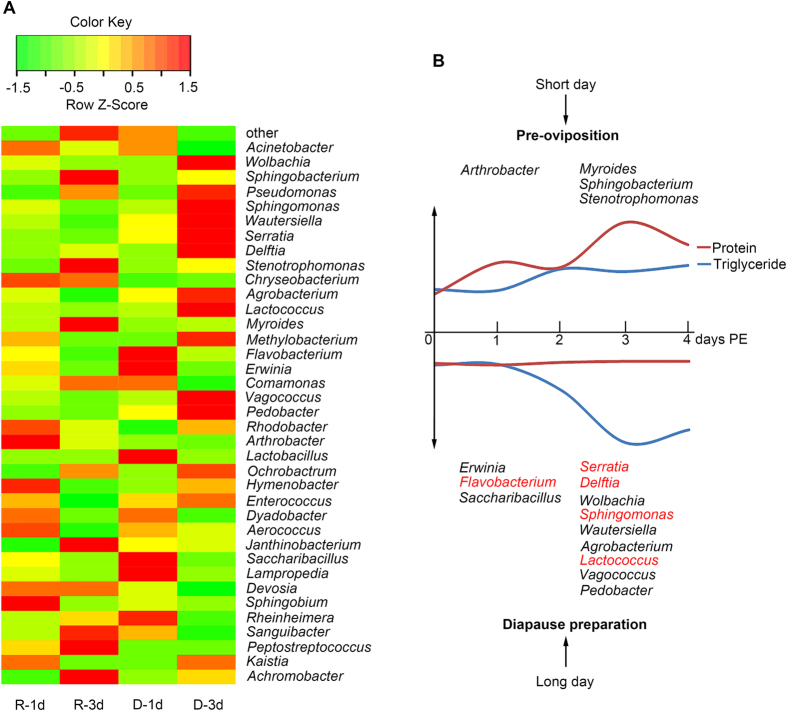
The distribution of gut microbiota at the genus level during the four day post- eclosion period in reproductive and diapause-destined female *C. bowringi*. (**A**) Heatmap of the relative abundance of bacterial communities at the genus level in each treatment. (**B**) Model of genus differences (*P* < 0.05) in gut microbiota between reproductive and diapause-destined female *C. bowringi* at 25 °C. Red font denotes the gut microbiota associated with mammalian obesity and nutritional metabolism. Trends in various nutrients over the four day post-eclosion period were generated from data from previous work^4^.

We next analyzed the distribution of gut bacteria communities at the genus level to infer the specific function of microbiota in reproduction and diapause (Fig. 5A). *Arthrobacter* (*P* = 0.045) was the dominant genus in the R-1d group. However, *Myroides* (*P* = 0.003), *Sphingobacterium* (*P* = 0.036), and *Stenotrophomonas* (*P* = 0.045) were the main genera in the R-3d group. These results suggest that these genera may be involved in the reproductive development of *C. bowringi*. There is evidence that an endocellular bacterial symbiont, *Buchnera aphidicola*, can influence the reproduction of aphids by regulating host amino acid biosynthesis^25^,^26^. Gut bacteria communities also are essential for insects’ reproduction^27^,^28^. Therefore, the specific gut microbiota associated with reproductive development in this study may contribute to reproduction in *C. bowringi*.

The proportion of *Flavobacterium* (*P* = 0.002), *Saccharibacillus* (*P* = 0.006), and *Erwinia* (*P* = 0.004) were highest in the D-1d group (Fig. 5A). However, at the key stage for pre-diapause lipid accumulation (D-3d), *Wautersiella* (*P* = 0.018), *Pedobacter* (*P* = 0.031), *Vagococcus* (*P* = 0.041), *Lactococcus* (*P* = 0.020), *Delftia* (*P* = 0.036), *Serratia* (*P* = 0.044), *Agrobacterium* (*P* = 0.009), *Sphingomonas* (*P* = 0.041), and *Wolbachia* (*P* = 0.029) were the dominant genera. Although our results suggest that *Bacteroidetes*, *Firmicutes*, and *Proteobacteria* are associated with diapause, at the genus level these microbiota seem to regulate both reproduction and diapause. Nonetheless, *Flavobacterium,* which has been identified as obesity-associated gut microbiota in mammals^20^,^24^, were associated with diapause (*P* < 0.05). In addition, obesity-related microbiota, including *Lactococcus*^29^ (*P* = 0.020), *Delftia*^30^ (*P* = 0.036), *Serratia*^31^ (*P* = 0.044), and *Sphingomonas*^32^ (*P* = 0.041), were also more abundant in diapause-destined female *C. bowringi*. In summary, most of the gut microbiota associated with diapause in *C. bowringi* are the same as those associated with obesity in mammals.

A schematic representation of the association between gut microbiota genera and the nutritional metabolism of reproductive and diapause-destined *C. bowringi* is shown in Fig. 5B. Based on previous studies of mammalian obesity and insect nutritional metabolism, we think that triglyceride accumulation in diapause-destined female adults is associated with specific genera of gut microbiota. Although further study is needed to verify the exact relationships between bacterial genera and lipid accumulation, we believe that the results presented in this paper may open a new avenue for studying insect diapause, and also allow the development of a natural insect obesity model with which to exploring the mechanisms of human obesity. In addition, given the fact that diapause is controlled by the endocrine system^33^, we speculate that gut microbiota may interact with hormone signaling pathways to regulate diapause. Further research on diapause, as well as that on human obesity and metabolic disorders, should take into account the possibility of such communication between the endocrine system and gut microbiota.

## Methods

### Insect rearing

*C. bowringi* were originally collected from Xiushui County (29°10 N, 114°40E), Jiangxi Province, China, and maintained in our laboratory on radish, *Raphanus sativus* L. var. *longipinnatus* (Brassicales: Brassicaceae)^34^. We obtained nondiapause-destined (reproductive) adults by rearing larvae at 25 °C under a 12:12 h light:dark photoperiod, and diapause-destined adults by rearing larvae at 25 °C under the 16:8 h light:dark photoperiod^16^. During the 4 day PE period, proteins and carbohydrates are stored in the ovary of reproductive females, while lipids (triglycerides) are accumulated in the fat body of diapause-destined females^4^. Hence, we hypothesized that different gut microbiota may be involved in each case, and collected samples from both reproductive and diapause-destined females at 1 and 3 days PE (Fig. 1A).

### Gut microbiota collection, DNA extraction and sequencing

We set up four experimental treatments, including R-1d, R-3d, D-1d, and D-3d. Each treatment had four independent biological replicates, and each replicate required a mixture of gut samples from 20 adult females. To Prior to dissection, the females were soaked in 70% ethanol for 3 minutes, and rinsed three times in sterile phosphate-buffered saline. Gut samples were dissected from females with sterilized tweezers and eye scissors under a stereomicroscope. During dissection, we also observed ovarian development to confirm the reproductive, or pre-diapause, status of females as appropriate. Total metagenomic DNA were extracted and purified from gut samples with a Universal Genomic DNA Kit (CwBio, Inc., Beijing, China) according to the manufacturer’s instructions. The concentration of metagenomic DNA was measured using Qubit Platform (Life Technologies, CA, USA). The V3-V4 hypervariable region of 16S ribosomal RNA gene was amplified from 30 ng of each metagenomic DNA in triplicate polymerase chain reactions (PCR) with Pyrobest DNA polymerase (TaKaRa, Dalian, China) and the forward and reverse primers 5′-ACTCCTACGGGAGGCAGCAG-3′ and 5′-GGACTACHVGGGTWTCTAAT-3′^35^ (Sangon Biotech, Shanghai, China), following the manufacturer’s instructions. Barcode sequences (see Supplementary Table S2) were attached to the amplification primers to distinguished the different samples. PCR products were viewed on 2% agarose electrophoresis gel and the respective amplicon libraries generated. Gut microbial DNA was then sequenced by Honortech (Beijing, China) using the Illumina MiSeq platform.

### Bioinformatics statistics

Based on the raw data (The Sequence Read Archive accession: SRP078307), pair-end reads were spliced using the principle of 98% overlap of 19 bases using Connecting Overlapped Pair-End software^36^. Barcode and primer sequences were then filtered to obtain clean data. Operational Taxonomic Unit (OTU) generation and clustering were done with USEARCH^37^ on the basis of 97% identity. Singletons were not used for clustering, only for OTU quantification. Core microbiota was identified with QIIME^38^. UCLUST^37^ and GreenGenes^39^ were used to perform species annotation of OTUs under a similarity threshold of 90–100%. The same sequencing depth was used to compare the alpha-diversity, beta-diversity, and the abundance of 16S metagenome data. Species diversity was determined by drawing a rarefaction curve and PCoA analysis with QIIME^38^. The relative abundance of gut microbiota was calculated according to the species annotation and reads number. A heatmap of the relative abundance of gut microbiota at the genus level was generated using custom Perl scripts. The transformed data of relative abundance were based on the Z-scores, which were calculated by the following formula.


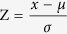


The *x* is a raw score, *μ* is the mean of the population, and *σ* is the standard deviation of the population. We analyized the significant difference between the two groups of samples with different taxonomic levels using metastats^40^. “*P*” value shows the test of significance, and *P* < 0.05 indicates the significant difference.

## Additional Information

**How to cite this article**: Liu, W. *et al*. Association between gut microbiota and diapause preparation in the cabbage beetle: a new perspective for studying insect diapause. *Sci. Rep.* **6**, 38900; doi: 10.1038/srep38900 (2016).

**Publisher's note:** Springer Nature remains neutral with regard to jurisdictional claims in published maps and institutional affiliations.

## Supplementary Material

Supplementary Information

Supplementary Dataset 1

## Figures and Tables

**Table 1:** The 17 core microbiota at the genus level in the gut of nondiapause- and diapause-destined *C. bowringi* during the 4 days post-eclosion.

OTUs	Genera
OTU2	*Acinetobacter*
OTU3	*Wolbachia*
OTU6	*Enterobacter*
OTU7	*Delftia*
OTU9	*Pseudomonas*
OTU10	*Stenotrophomonas*
OTU11	*Agrobacterium*
OTU12	*Wautersiella*
OTU13	*Sphingobacterium*
OTU14	*Chryseobacterium*
OTU17	*Serratia*
OTU20	*Sphingomonas*
OTU21	*Rhodoferax*
OTU28	*Methylobacterium*
OTU41	*Citrobacter*
OTU42	*Arthrobacter*
OTU45	*Vagococcus*
