# Supplementary material for: Association between gut microbiota and diapause preparation in the cabbage beetle: a new perspective for studying insect diapause
Source: Sci Rep. 2016 Dec 9;6:38900. doi: 10.1038/srep38900 (PMC5146961; doi:10.1038/srep38900)
Supplement: Supplementary Information [file srep38900-s1.doc]

Association between gut microbiota and diapause preparation in the cabbage beetle: a new perspective for studying insect diapause

Wen Liu, Yi Li, Shuang Guo, Han Yin, Chao-Liang Lei, Xiao-Ping Wang*

**Supplementary Table S2. Barcode sequences used for distinguishing the different samples**

| **Samples** | **P7 end barcodes** | **P5 end barcodes** |
| --- | --- | --- |
| R-1d replicate 1 | GCTTACGA | TAATTACC |
| R-1d replicate 2 | CGTGACGG | TAATTACC |
| R-1d replicate 3 | TACTTCGC | TAATTACC |
| R-1d replicate 4 | CGCAGTCC | TAATTACC |
| R-3d replicate 1 | TGAACCTC | TAATTACC |
| R-3d replicate 2 | TTGTACTC | TAATTACC |
| R-3d replicate 3 | CAATGCTC | TAATTACC |
| R-3d replicate 4 | CACGGCGA | TAATTACC |
| D-1d replicate 1 | CGCCGCTG | TAATTACC |
| D-1d replicate 2 | GCATCCTT | TAATTACC |
| D-1d replicate 3 | GCCATTGC | TAATTACC |
| D-1d replicate 4 | GAGAATAC | TAATTACC |
| D-3d replicate 1 | GTAATGAC | TAATTACC |
| D-3d replicate 2 | GCTTGGAT | TAATTACC |
| D-3d replicate 3 | TTAACACA | TAATTACC |
| D-3d replicate 4 | CGGCACCT | TAATTACC |

Note: P7 and P5 are adaptors used to link DNA segments with chip.
